# The impact of an unknown HIV serostatus on inpatient mortality

**DOI:** 10.11604/pamj.2017.28.285.11860

**Published:** 2017-11-30

**Authors:** Yoshan Moodley

**Affiliations:** 1 Discipline of Anaesthesiology and Critical Care Medicine, Nelson R Mandela School of Medicine, University of KwaZulu-Natal, South Africa

**Keywords:** HIV, unknown serostatus, hospital mortality

## Abstract

**Introduction:**

Determining HIV serostatus is crucial for linking HIV-infected patients to appropriate care, which might reduce their risk of subsequent morbidity and mortality. A recent South African study demonstrated a potentially harmful association between an unknown HIV serostatus and rehospitalisation. The impact of an unknown HIV status on inpatient mortality has not yet been established in that setting, which formed the impetus for the current study.

**Methods:**

This was an unmatched case-control analysis of adult patient data collected as part of a demographic survey at the Hlabisa Hospital, South Africa between October 2009 and February 2014. Cases were defined as patients who suffered inpatient mortality, while controls were patients who did not suffer inpatient mortality. A sample size of 92 cases and 276 controls was used in this study. Patient data related to age, gender, distance between referral clinic and the hospital, HIV serostatus (HIV-negative, HIV-positive or an unknown HIV serostatus) and comorbidity were analysed using recommended methods for unmatched case-control studies.

**Results:**

When potential confounders were accounted for, we found an unknown HIV serostatus to be associated with an almost 8-fold increase in the odds of inpatient mortality when compared with patients who were known HIV-negative (Odds Ratio: 7.64, 95% Confidence Interval: 1.11-52.33, p = 0.038).

**Conclusion:**

An unknown HIV serostatus was independently associated with a higher odds of inpatient mortality. This finding highlights the potential benefit of adopting an “opt-out” approach to HIV counseling and testing. Further research on this topic is required.

## Introduction

The Southern African region continues to be disproportionately burdened by HIV/AIDS. South Africa is one of the most severely afflicted countries in this region [1]. Despite the estimated prevalence of HIV in the population ranging between 11% and 20%, half of all adult South Africans do not known their HIV serostatus [2]. Determining HIV serostatus is crucial for linking HIV-infected patients to appropriate care, which might reduce their risk of subsequent morbidity and mortality [3, 4]. Large clinical studies investigating patient outcomes (including mortality) are scarce in resource-limited settings due to logistical challenges in creating and maintaining patient registries at healthcare facilities [5]. This has since improved with the establishment of surveillance mechanisms, such as the publically-available Africa Centre Demographic Information System (ACDIS), which also collected inpatient data from a hospital located in a South African setting with a high prevalence of HIV infection [6]. A recent analysis of this hospital data collected as part of ACDIS suggested a statistical trend toward a potentially harmful association between an unknown HIV serostatus and rehospitalisation [7]. The impact of an unknown HIV serostatus on inpatient mortality in the same setting is yet to be established. An understanding of any potential association between an unknown HIV serostatus and inpatient mortality might have important implications on initiatives aimed at improving the uptake of HIV counseling and testing in this setting, as well as improving subsequent linkage to care in patients with previously undiagnosed HIV infection. This formed the impetus for the current study.

## Methods

**Study design, setting and study population**: We performed an unmatched case-control analysis of adult medical inpatient data collected between October 2009 and February 2014 at the Hlabisa Hospital in KwaZulu-Natal Province, South Africa as part of ACDIS [6]. The hospital is considered an important primary care facility in the region, serving a predominantly rural population. HIV incidence in the Hlabisa district of KwaZulu-Natal has been estimated at 3.2/100 person years [6]. The methodology suggested by Breslow et al. was used to conduct this unmatched case-control study [8].

**Case and control definitions**: Cases were defined as patients who suffered mortality while hospitalized. Inpatient mortality was determined from the patient discharge disposition variable in the ACDIS Hlabisa Hospital dataset. Controls were defined as patients who did not suffer mortality while hospitalized.

**Sample size calculation**: The sample size required for this study was 368 (92 cases and 276 controls) patients. The following parameters were used to calculate the study sample size: anticipated odds ratio -2.0, estimated exposure of controls -25% (anticipated to be lower than the 50% reported for the general South African adult population as it was likely that patient HIV serostatus might have been determined at other healthcare facilities), alpha risk -5%, power -80%, and a case:control ratio of 1:3. The case pool was determined to consist of 1 509 patients, while the control pool consisted of 9, 853 patients. Cases and controls were then randomly selected from each pool until the required sample of cases and controls was achieved.

**Patient demographics, HIV serostatus and clinical comorbidities**: Data related to age, gender and HIV serostatus (HIV-positive, HIV-negative or an unknown HIV serostatus) were extracted from the ACDIS Hlabisa Hospital dataset for each case and control. International Classification of Disease-10^th^ Revision (ICD-10) codes proposed by Quan et al were used to identify the presence of the following potentially important comorbidities: hypertension, diabetes, cancer, cardiovascular disease (a composite of myocardial infarction, stroke and heart failure) and renal disease [9]. ICD-10 codes for tuberculosis and pneumonia were adopted from those used by the Healthcare Cost and Utilization Project [10]. We also considered that the distance between the referral clinic and the hospital might also be an important factor impacting inpatient outcomes and we also included this variable in our final data analysis.

**Statistical analysis**: Case-control data were initially analyzed using X^2^, Fisher's Exact or Mann-Whitney tests where appropriate. Results for the aforementioned crude data analyses are presented as frequencies with percentages or medians with interquartile ranges (IQR). An unconditional logistic regression model was used to account for potential confounding in the unmatched case-control study design, with the results of this adjusted statistical analysis being presented as odds ratios (OR) with 95% confidence intervals (95% CI). A p-value of <0.05 was considered statistically significant. The Statistical Package for the Social Sciences (SPSS) version 24.0 (IBM Corp., USA) was used to perform all statistical analyses.

**Study ethics**: This study was approved by the Biomedical Research Ethics Committee of the University of KwaZulu-Natal, South Africa (Protocol EXM277/15).

## Results

**Descriptive statistics and crude analyses:** Results of the descriptive and crude statistical analyses are presented in Table 1. HIV serostatus was known in 51.9% of the study population (cases and controls combined), with 10.3% of patients being diagnosed as HIV-negative and 41.6% of patients being diagnosed as HIV-positive. Approximately 4 in every 10 patients were male. The median age of the study population was 34.0 (IQR: 26.0-49.0) years old. The median distance from the patient's referral clinic to the Hlabisa hospital was 47.3 (IQR: 27.9-50.4) kilometers. Tuberculosis was the most important comorbidity identified in the study population, afflicting almost a quarter of all patients. When crude comparisons were made between case and control groups, associations between the following clinical characteristics and mortality were noted: HIV serostatus (p < 0.001), increasing age (p < 0.001), pneumonia (p = 0.013), diabetes (p = 0.033), cardiovascular disease (p = 0.026), and tuberculosis (p < 0.001). The associations between the remaining clinical characteristics and mortality failed to reach statistical significance.

**Table 1:** Descriptive statistics and results of the crude statistical analyses expressed as frequencies (%) or medians (IQR)

Patient/Clinical Characteristic	Sub-Category	All patients (n = 368)	Cases (n = 92)	Controls (n = 276)	p-value
HIV serostatus					< 0.001
	HIV-Negative	38 (10.3)	2 (2.2)	36 (13.0)	
	HIV-Positive	153 (41.6)	61 (66.3)	92 (33.3)	
	HIV-unknown	177 (48.1)	29 (31.5)	148 (53.6)	
Median age in years	N/A	34 (26.0-49.0)	40 (31.0-54.8)	33 (25.0-46.0)	< 0.001
Gender					0.076
	Female	217 (59.0)	47 (51.1)	170 (61.6)	
	Male	151 (41.0)	45 (48.9)	106 (38.4)	
Median distance from referral clinic to hospital in kilometers	N/A	47.3 (27.950.4)	47.3 (8.0-50.4)	47.3 (27.9-50.4)	0.995
Pneumonia					0.013
	No	356 (96.7)	85 (92.4)	271 (98.2)	
	Yes	12 (3.3)	7 (7.6)	5 (1.8)	
Hypertension					0.595
	No	348 (94.6)	86 (93.5)	262 (94.9)	
	Yes	20 (5.4)	6 (6.5)	14 (5.1)	
Diabetes					0.033
	No	352 (95.7)	84 (91.3)	268 (97.1)	
	Yes	16 (4.3)	8 (8.7)	8 (2.9)	
Cancer					0.261
	No	364 (98.9)	90 (97.8)	274 (99.3)	
	Yes	4 (1.1)	2 (2.2)	2 (0.7)	
Cardiovascular disease					0.026
	No	360 (97.8)	87 (94.6)	273 (98.9)	
	Yes	8 (2.2)	5 (5.4)	3 (1.1)	
Tuberculosis					< 0.001
	No	279 (75.8)	45 (48.9)	234 (84.8)	
	Yes	89 (24.2)	47 (51.1)	42 (15.2)	
Renal disease					0.081
	No	356 (96.7)	86 (93.5)	270 (97.8)	
	Yes	12 (3.3)	6 (6.5)	6 (2.2)	

IQR: Interquartile Range, N/A: Not Applicable

**Adjusted analysis**: The results of the regression analysis are shown in Table 2. When compared with the HIV-negative serostatus patient group, patients in the HIV-positive serostatus and HIV-unknown serostatus groups experienced a significantly higher odds of suffering inpatient mortality (OR: 17.76, 95% CI: 2.70-116.92, p = 0.003; and OR: 7.64, 95% CI: 1.11-52.33, p = 0.038). There was a 2% increase in the odds of inpatient mortality per year increase in age (OR: 1.02, 95% CI: 1.0-1.04, p = 0.026). The remaining sociodemographic variables (male gender and distance between the referral clinic and the hospital) were not independently associated with a higher odds of inpatient mortality. Of the seven clinical comorbidities investigated in this study, only tuberculosis (OR: 6.54, 95% CI: 3.19-13.40, p < 0.001), renal disease (OR: 5.84, 95% CI: 1.29-26.35, p = 0.022) and diabetes (OR: 6.19, 95% CI: 1.69-22.65, p = 0.006) were found to be independent predictors of inpatient mortality.

**Table 2:** Results of the adjusted statistical analysis

Patient/Clinical Characteristic	Sub-Category	OR (95% CI)	p-value
HIV serostatus	HIV-Negative	Reference	-
	HIV-Positive	17.76 (2.70-116.92)	0.003
	HIV-unknown	7.64 (1.11-52.33)	0.038
Age (per year increase)	N/A	1.02 (1.00-1.04)	0.026
Gender	Female	Reference	-
	Male	0.96 (0.51-1.79)	0.891
Distance from referral clinic to hospital (per kilometer increase)	N/A	0.99 (0.97-1.00)	0.073
Pneumonia	No	Reference	-
	Yes	3.46 (0.91-13.15)	0.068
Hypertension	No	Reference	-
	Yes	1.03 (0.28-3.85)	0.968
Diabetes	No	Reference	-
	Yes	6.19 (1.69-22.65)	0.006
Cancer	No	Reference	-
	Yes	8.57 (0.94-77.71)	0.056
Cardiovascular disease	No	Reference	-
	Yes	6.40 (0.69-58.99)	0.102
Tuberculosis	No	Reference	-
	Yes	6.54 (3.19-13.40)	< 0.001
Renal disease	No	Reference	-
	Yes	5.84 (1.29-26.35)	0.022

OR: Odds Ratio, CI: Confidence Interval, N/A: Not Applicable

## Discussion

When potential confounders were accounted for, we found an unknown HIV serostatus to be associated with an almost 8-fold increase in the odds of inpatient mortality when compared with patients who were known HIV-negative. The odds of inpatient mortality in the unknown HIV serostatus group was almost 2.5 times lower than that observed for the known HIV-positive serostatus group. Our findings of a high odds of inpatient mortality in the HIV-positive serostatus group are to be expected. In overseas settings HIV infection is still considered an important contributor to poor inpatient outcomes, as evidenced by its inclusion in comorbidity measures such as the Charlson Comorbidity Index [11]. In African settings, high levels of inpatient mortality in the HIV-infected population have been attributed to several operational and clinical challenges which the continent faces [12]. When viewed in context with the findings for the HIV-positive serostatus group, our findings for the unknown HIV serostatus group suggest that while there might be a proportion of the unknown HIV serostatus population who upon HIV counseling and testing will prove to be HIV-negative, a larger proportion of the unknown HIV serostatus population are undiagnosed HIV-positive. This would explain the harmful association between an unknown HIV serostatus and inpatient mortality which was observed in this study. These findings highlight the importance of HIV counseling and testing initiatives in settings with a high prevalence of HIV infection. Currently, a systematic “opt-out" approach to HIV counseling and testing is used to target specific South African patient populations, such as those patients attending antenatal, tuberculosis or sexually transmitted infection clinics [13]. With this approach there are a number of missed opportunities to identify undiagnosed HIV-positive patients who do not fall in any of the aforementioned targeted patient groups but are still in need of antiretroviral therapy which might improve survival. While the “opt-out” approach would ultimately reduce the number of undiagnosed HIV-infected patients, this approach does pose certain challenges in a resource-limited setting such as the Hlabisa Hospital. Firstly, more healthcare workers would require training in HIV counseling and testing.

Secondly, there need to be measures in place to ensure that facilities have access to HIV testing kits and no kit stock outs occur. Thirdly, care structures to which newly diagnosed patients are referred must be improved and prepared for an increase in patient volumes. Importantly, these challenges will result in an increase in healthcare expenditure and would require careful allocation of healthcare resources. Careful planning and consultation amongst public health specialists would be required to overcome these potential challenges for an “opt-out” approach to HIV counseling and testing to be viable in our setting. While we found several other patient/clinical characteristics to be independently associated with/not independently associated with a higher odds of inpatient mortality, these findings should be interpreted with caution. The sample size used in this study was derived with specific reference to determining the impact of HIV serostatus on inpatient mortality, while other characteristics were included to merely adjust for potential confounding during the unconditional logistic regression analysis. Therefore, it is likely that this study was not adequately powered to investigate the impact of several of the other patient/clinical characteristics mentioned above. Further case-control analyses (with appropriately revised sample sizes) for each patient/clinical characteristic of interest would be required to draw conclusions related to the impact of these patient/clinical characteristics on inpatient mortality. There were additional limitations to our study. Firstly, this study was an analysis of data from a single rural healthcare facility. This entails that the findings of this study might not necessarily be generalizable to other healthcare facilities. A larger multicenter study would be required to confirm our findings. Secondly, only inpatient mortality was investigated in this research. While 30-day mortality would be a more appropriate outcome, patient loss to follow-up is a major challenge in resource-limited settings [14]. Strategies for estimating 30-day mortality in medical patients in these settings are required. Nevertheless, our use of inpatient mortality as our study outcome is justified as this outcome is an important outcome used in healthcare facilities [15]. Lastly, data related to medication use was not collected as part of the hospital database and we were unable to adjust for the effects of medication use in our logistic regression model. Prospective research is required to investigate the potential impact of medication use on inpatient mortality in our setting.

## Conclusion

In conclusion, we found that when compared with a HIV-negative serostatus, an unknown HIV serostatus was associated with an almost 8-fold increase in the odds of suffering inpatient mortality in our setting. It is possible that a shift toward a systematic “opt-out” approach to HIV counseling and testing in our setting might be useful in identifying undiagnosed HIV-positive patients and initiating these patients on antiretroviral therapy for improved survival. However, this would require careful planning to ensure its viability. Further research is required to confirm our study findings.

**What is known about this topic**

- Large clinical studies investigating HIV patient outcomes (including inpatient mortality) are scarce in resource-limited settings;
- An unknown HIV serostatus is associated with rehospitalisation in a South African setting.

**What this study adds**

- This study provides a report of the association between an unknown HIV serostatus and inpatient mortality in a South African setting;
- This study presents data highlighting the need for a systematic “opt-out” approach to HIV counseling and testing in South Africa.

## Competing interests

The author declares no competing interests.

## References

[1] Williams BG, Gouws E, Somse P, Mmelesi M, Lwamba C, Chikoko T (2015). Epidemiological Trends for HIV in Southern Africa: implications for reaching the elimination targets. Curr HIV/AIDS Rep..

[2] Kalichman SC, Simbayi LC (2003). HIV testing attitudes, AIDS stigma, and voluntary HIV counselling and testing in a black township in Cape Town, South Africa. Sex Transm Infect..

[3] Aziz M, Smith KY (2011). Challenges and successes in linking HIV-infected women to care in the United States. Clin Infect Dis..

[4] Branson BM, Handsfield HH, Lampe MA, Janssen RS, Taylor AW, Lyss SB (2006). Revised recommendations for HIV testing of adults, adolescents and pregnant women in health-care settings. MMWR Recomm Rep..

[5] O'Brien KS, Soliman AS, Awuah B, Jiggae E, Osei-Bonsu E, Quayson S (2013). Establishing effective registration systems in resource-limited settings: cancer registration in Kumasi, Ghana. J Registry Manag..

[6] Tanser F, Hosegood V, Barnighausen T, Herbst K, Nyirenda M, Muhwava W (2008). Cohort Profile: Africa centre Demographic Information System (ACDIS) and population-based HIV survey. Int J Epidemiol..

[7] Moodley Y, Tomita A (2017). Relationship between HIV serostatus, CD4 count and rehospitalisation: Potential implications for health systems strengthening in South Africa. S Afr J Infect Dis..

[8] Breslow NE, Day NE (1987). Statistical methods in cancer research: IARC Workshop 25-27 May 1983. IARC Sci Publ..

[9] Quan H, Sundararajan V, Halfon P, Fong A, Burnand B, Luthi JC (2005). Coding algorithms for defining comorbidities in ICD-9-CM and ICD-10 administrative data. Med Care..

[10] Steiner C, Elixhauser A, Schnaier J (2002). The healthcare cost and utilization project: an overview. Eff Clin Pract..

[11] Quan H, Li B, Couris CM, Fushimi K, Graham P, Hider P (2011). Updating and validating the Charlson comorbidity index and score for risk adjustment in hospital discharge abstracts using data from 6 countries. Am J Epidemiol..

[12] Wajanga BM, Webster LE, Peck RN, Downs JA, Mate K, Smart LR (2014). Inpatient mortality of HIV-infected adults in sub-Saharan Africa and possible interventions: a mixed methods review. BMC Health Serv Res..

[13] Clouse K, Hanrahan CF, Bassett J, Fox MP, Sanne I, Van Rie A (2014). Impact of systematic HIV testing on case finding and retention in care at a primary care clinic in South Africa. Trop Med Int Health..

[14] Honge BL, Jespersen S, Nordentoft PB, Medina C, da Silva D, da Silva ZJ (2013). Loss to follow-up occurs at all stages in the diagnostic and follow-up period among HIV-infected patients in Guinea-Bissau: a 7-year retrospective cohort study. BMJ Open..

[15] Franklin PD, Legault JP (1999). Using data to evaluate hospital inpatient mortality. J Nurs Care Qual..

